# Erratum: Parvalbumin-expressing interneurons coordinate hippocampal network dynamics required for memory consolidation

**DOI:** 10.1038/ncomms16120

**Published:** 2017-07-06

**Authors:** Nicolette Ognjanovski, Samantha Schaeffer, Jiaxing Wu, Sima Mofakham, Daniel Maruyama, Michal Zochowski, Sara J. Aton

Nature Communications 8: Article number: 15039; DOI: 10.1038/ncomms15039 (2017); Published: 04 06 2017; Updated: 07 06 2017

The original version of the Supplementary Information attached to this Article did not contain Supplementary Figures 11–16 and Supplementary References. The HTML has now been updated to include a corrected version of the Supplementary Information file.

## Supplementary Material

Supplementary Information Supplementary Figures and Supplementary References

