# Supplementary material for: Parvalbumin-expressing interneurons coordinate hippocampal network dynamics required for memory consolidation
Source: Nat Commun. 2017 Apr 6;8:15039. doi: 10.1038/ncomms15039 (PMC5384212; doi:10.1038/ncomms15039)
Supplement: Supplementary Information — Supplementary Figures and Supplementary References [file ncomms15039-s1.pdf]

Supplementary Figure 1

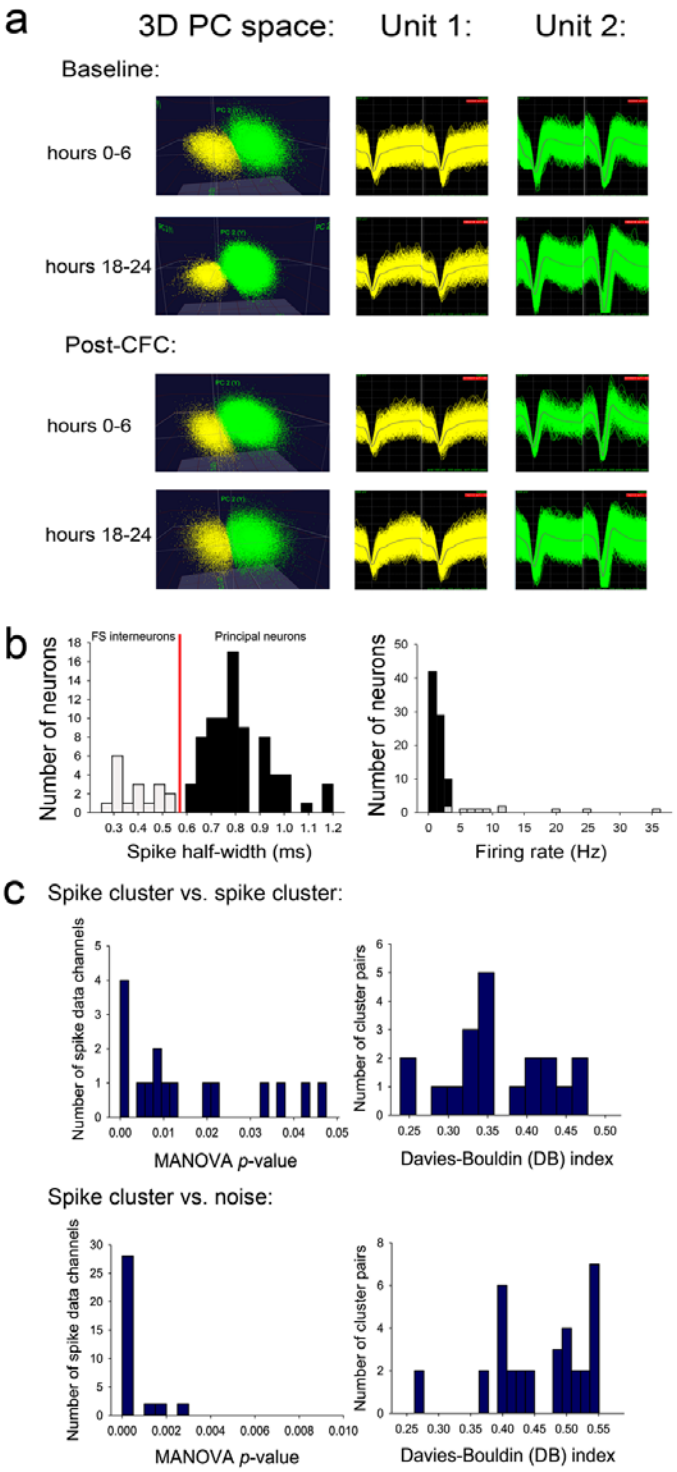

Supplementary Figure 1: Spike sorting for recordings from freely-behaving mice. (**a**) An example of spike cluster separation vs. time across baseline and post-CFC recording period. (**b**) Distribution of spike half-widths (in ms; *left*) and mean waking firing rates (*right*) for all stably-recorded neurons with data shown in **Fig. 1**. Red line indicates threshold value for classification as FS interneurons (light gray; half-widths ≤ 0.55 ms) or principal neurons (black; half-widths > 0.55 ms). Mean half-widths (± SEM) for FS interneurons and principal neurons were 0.39 ± 0.03 and 0.81 ± 0.02 ms, respectively (comparable to previously published measurements for CA1 FS interneurons and pyramidal neurons). [1,2] Mean firing rates (± SEM) for FS interneurons (light gray) and principal neurons (black) were 9.9 ± 2.6 and 1.2 ± 0.1 Hz, respectively. Both spike half-width distributions and firing rate distributions were significantly different between the two cell populations (p < 0.001, Mann-Whitney U test). (**c**) Spike cluster quality metrics for individual spike clusters vs. one another (*top*) and vs. noise (*bottom*). *Left:* Distributions of MANOVA p-values for principal component-based spike cluster separation, for all channels with multiple stably-recorded neurons. *Right:* Distributions of Davies-Bouldin (DB) validity indices for cluster pairs on channels with multiple stably-recorded neurons.

Supplementary Figure 2

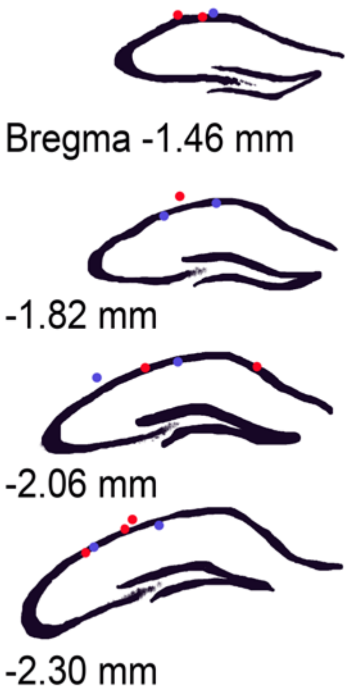

Supplementary Figure 2: Recording sites for C57BL/6J mice from experiments shown in Fig. 1. Positions of electrode bundles for single-unit and LFP recordings for C57BL/6J mice used in CFC (*n* = 4 mice; red dots) and sham (*n* = 4 mice; purple dots) experiments.

Supplementary Figure 3

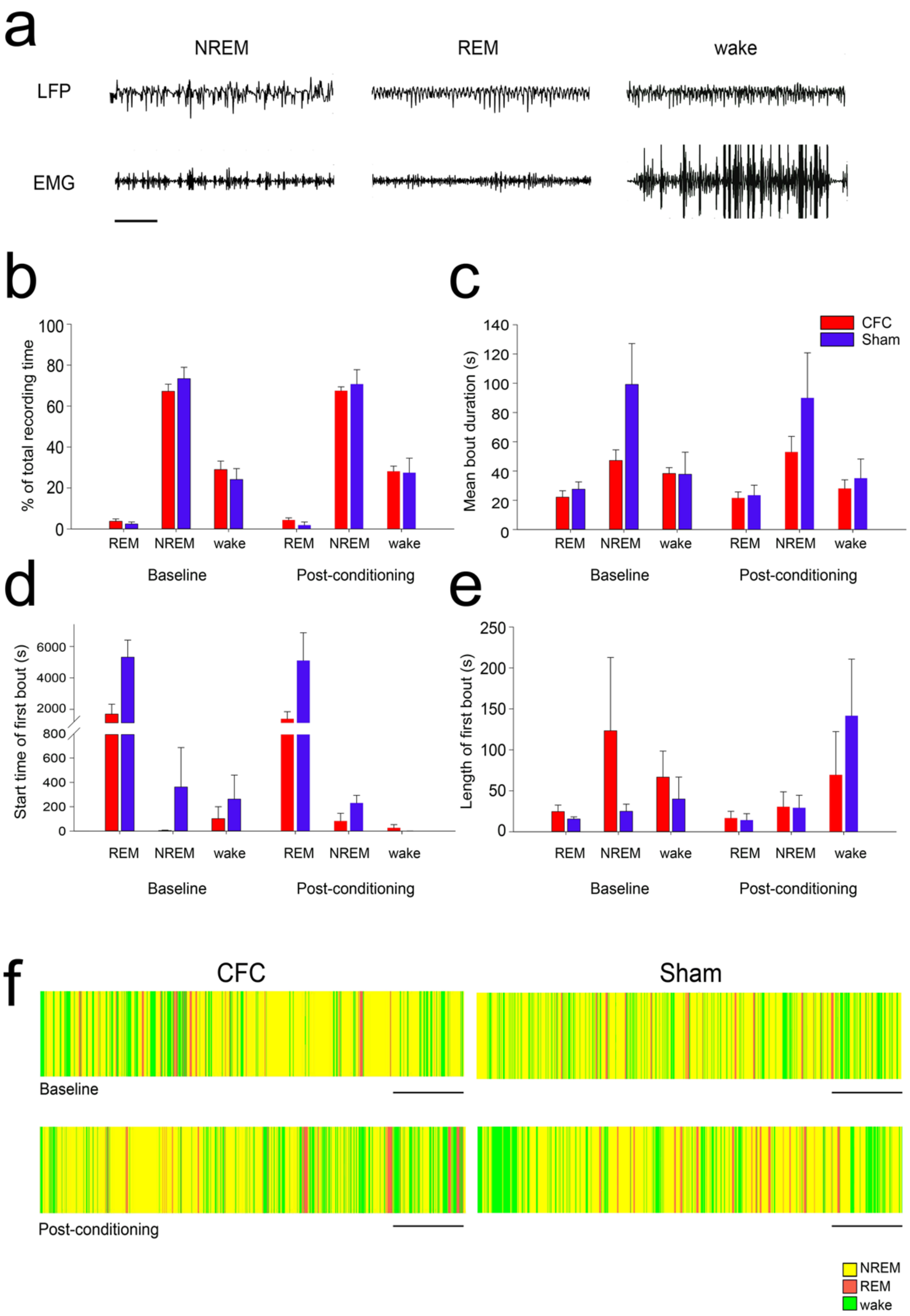

Supplementary Figure 3: Sleep architecture in C57BL/6J mice. Representative LFP and EMG traces in (**a**) show typical activity during NREM, REM, and wake. Scale bar = 2 s. There was no significant effect of treatment on (**b**) percent of time spent in REM, NREM, and wake, (**c**) mean duration of REM, NREM, and wake bouts, (**d**) time to onset of the first bout of REM, NREM, and wake, or (**e**) the length of the first bout of REM, NREM, and wake. All error bars are SEM. (**f**) Hypnograms showing sleep state transitions over the first 6 h of either baseline (***top***) or post-conditioning (***bottom***) recording, in representative CFC (***left***) and Sham (***right***) mice. Scale bars = 1 h.

Supplementary Figure 4

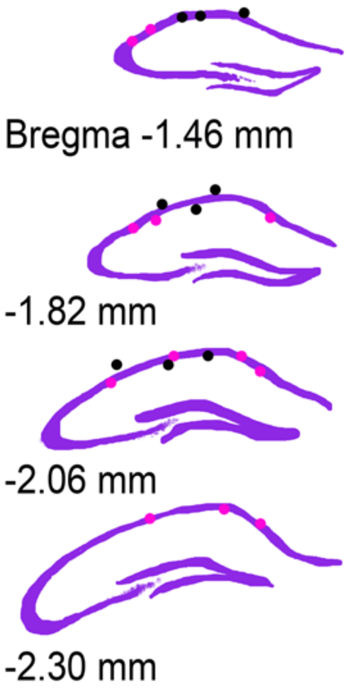

Supplementary Figure 4: Electrode placement in hM4Di- and mCherry-expressing mice. Positions of electrode bundles for single-unit and LFP recordings for *Pvalb-IRES-CRE* mice expressing either control virus (mCherry; *n* = 4 mice; black dots) or hM4Di-mCherry (*n* = 6 mice; pink dots).

Supplementary Figure 5

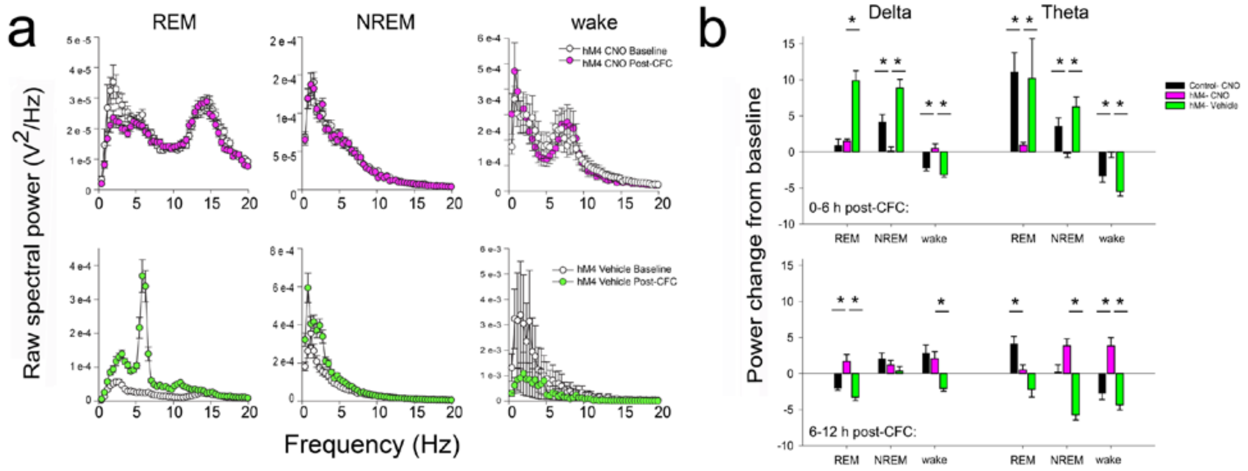

Supplementary Figure 5: LFP spectral changes across REM, NREM, and wake across hours 0-6 and 6-12 post-CFC. (**a**) Raw CA1 LFP spectral power in REM, NREM, and wake from the same hM4Di-expressing mouse shown in **Fig. 4** over the first 6 h post-CFC. Data are shown separately for baseline and following post-CFC administration of CNO (white and pink circles, respectively, top), and for a second baseline two weeks later and following post-CFC administration of vehicle (white and green circles, respectively, bottom). Post-CFC increases in delta and theta spectral power (from baseline) were present in hours 0-6 of REM and NREM sleep (but not wake) in the two control conditions. These increases were largely absent in hours 6-12 post-CFC. * indicates $p < 0.05$, Holm-Sidak *post hoc* test. Mean ± SEM shown for all LFP values across a given treatment.

Supplementary Figure 6

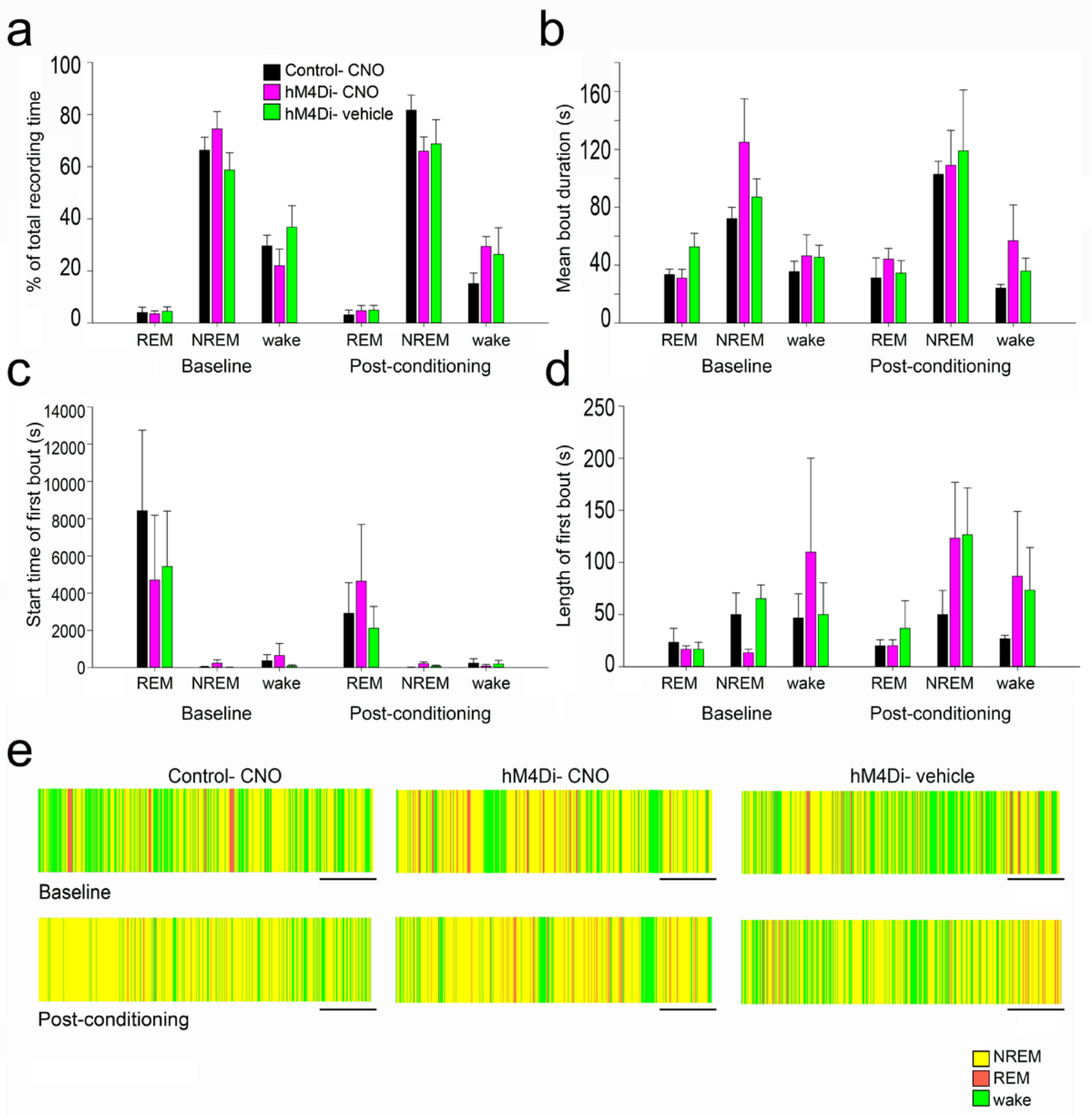

Supplementary Figure 6: Sleep architecture during pharmacogenetic experiments. There was no significant effect of treatment on either (**a**) percent of time spent in REM, NREM, and wake, (**b**) the mean duration of REM, NREM, and wake bouts, (**c**) time to onset of the initial bout of REM, NREM, and wake or (**d**) the length of initial bout of REM, NREM, and wake. All error bars are SEM. (**e**) Representative hypnograms for hours 0-6 at either baseline (***top***) or post-

conditioning (***bottom***) for Control-CNO (***left***), hM4Di-CNO (***center***), and hM4Di- vehicle (***right***) animals. Scale bars = 1 h.

Supplementary Figure 7

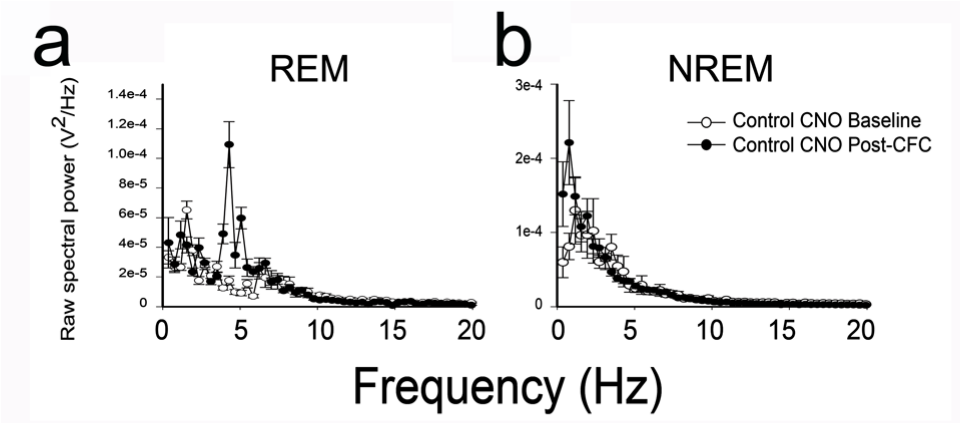

Supplementary Figure 7: CA1 LFP spectral power for mCherry-expressing mice. Raw CA1 LFP spectral power during the first 6 hours of recording at baseline (white circles) and post-CFC (black circles) for a representative *Pvalb-IRES-CRE* mouse expressing control (mCherry) virus during (**a**) REM and (**b**) NREM sleep (Mean ± SEM shown for local field potential values for all animals within treatment group hM4-vehicle, *n* = 74).

Supplementary Figure 8

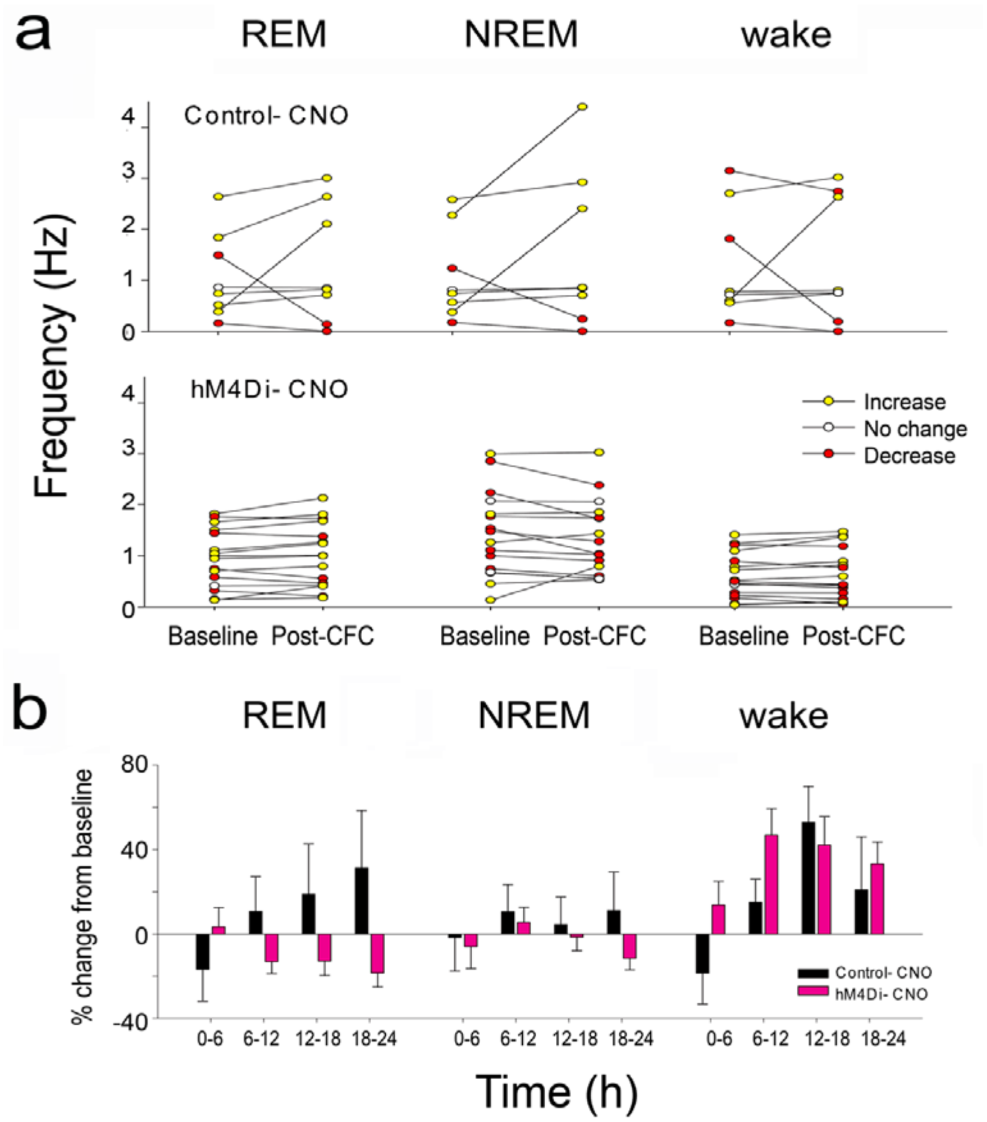

Supplementary Figure 8: CA1 firing rate changes following pharmacogenetic inhibition of PV+ interneurons. (**a**) Firing rate changes (in the first 6 h following CFC and CNO treatment) for all neurons stably recorded from a representative mouse in each treatment group (*n* = 8, Control- CNO; *n* = 15, hM4Di-CNO) . Increases and decreases in mean firing rate of ≥ 5% from the same period at baseline are shown in yellow and red, respectively. (**b**) In hM4Di-expressing

mice, CNO did not significantly affect mean firing rates in the first 6 h following administration. In mCherry-expressing control mice, a gradual increase in REM firing rates was observed over the 24 hours following CFC. This change was blocked by post-CFC administration of CNO to hM4Di-expressing mice (virus x time interaction $p < 0.05$, two-way RM ANOVA). Post-CFC firing rates during NREM sleep and wake were unaffected following CNO administration (NREM - *N.S.*, two-way RM ANOVA; wake - main effect of time: $p < 0.001$; virus x time interaction *N.S.*, two-way RM ANOVA). Mean ± SEM shown for individual cell firing rate for all animals within treatment group. $n = 27$, Control-CNO; $n = 35$, hM4Di-CNO.

Supplementary Figure 9

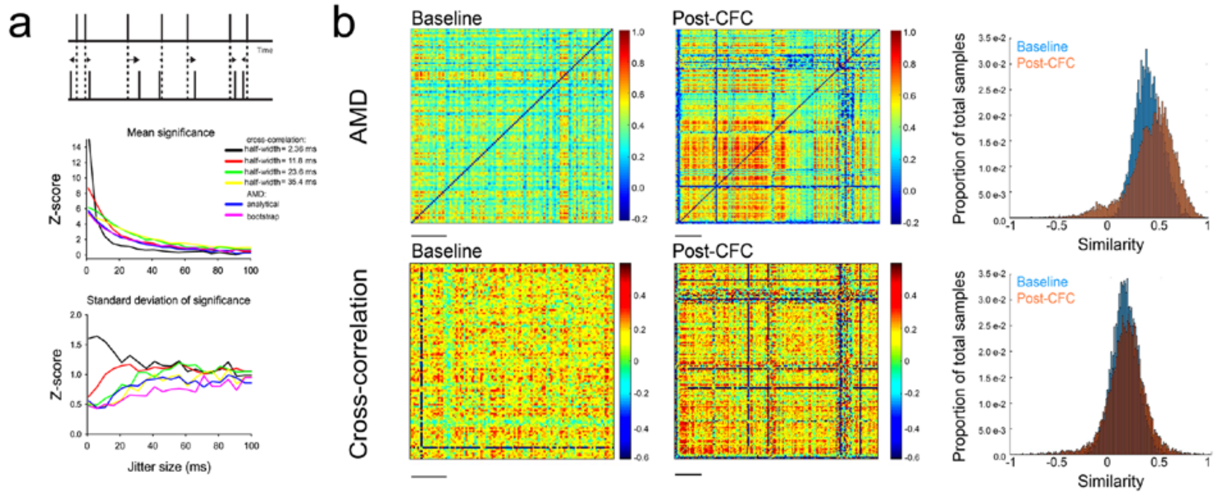

Supplementary Figure 9: Comparison of AMD-based and cross-correlation-based metrics for functional connectivity and stability analyses. (**a**) AMD and cross-correlation measures were compared for quantifying functional connectivity of two simulated spike trains. ***Top:*** The first 1-s, 30-spike train was generated randomly. The second train was generated by introducing jitter to the times of spikes from the first train. The amount of jitter was varied systematically between 1 and 100 ms, to determine how this variable (which should decrease with stronger functional connectivity) affects the two measures. ***Middle:*** Comparison of significance of functional connectivity measures for the two spike trains, across a range of jitter sizes. Cross-correlation significance between the two trains was calculated at 0 lag based on bootstrapping with 100 randomized spike trains, and Gaussian convolution of various half-widths. For comparison, AMD significance was calculated both analytically and using a similar bootstrapping technique (see **Methods**). ***Bottom:*** Standard deviation of functional connectivity significance values was estimated over 100 repetitions using randomized spike trains. Across a range of jitter sizes, AMD metrics provide a more reliable estimate of significance. (**b**) Comparison of AMD and cross-correlation for quantifying network stability using *in vivo* data.

AMD-based FSMs (***top***; data reproduced from **Fig. 7**) and cross-correlation-based FSMs (***bottom***) for the same network data recorded at baseline, and over the first 6 h post-CFC. Scale bar = 20 min. Distributions of minute-to-minute similarity values showed a greater degree of change after CFC when AMD was used to calculate functional connectivity.

Supplementary Figure 10

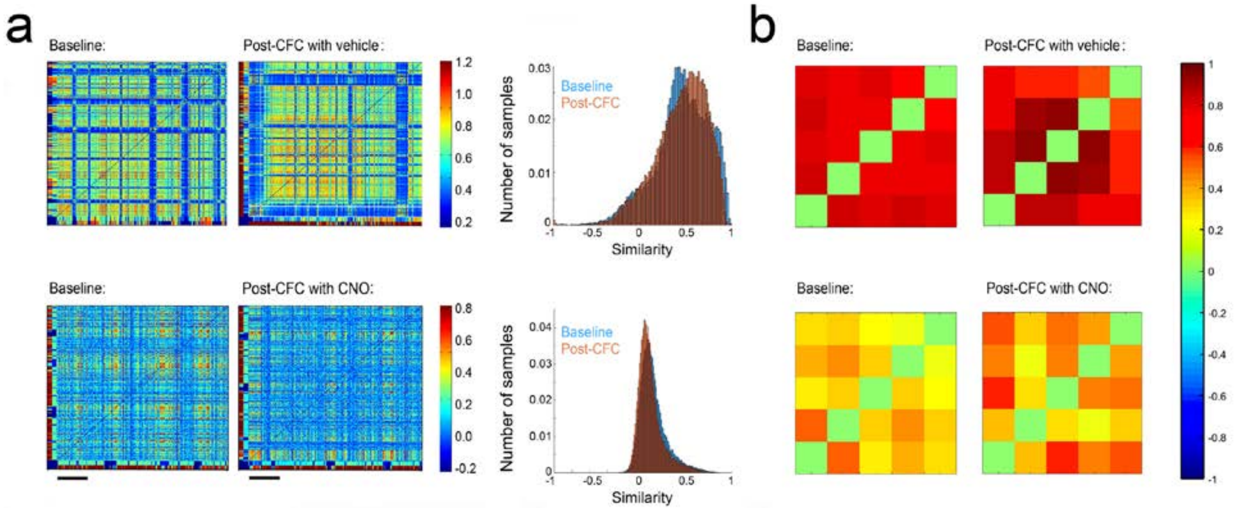

Supplementary Figure 10: FSMs for a representative mouse calculated across all behavioral states, and across only the longest intervals of NREM sleep.

(**a**) ***Left:*** CA1 network FSMs across all behavioral states, for the same mouse shown in **Fig. 7** at baseline, and over the first 6 h post-CFC (vehicle and CNO conditions). Scale bar = 1 h. Color bars along the outer edge of axes represents the behavioral state of the animal (blue - wake, red - NREM or REM, mixed sleep and wake are represented by color blends, *e.g.,* yellow) and inner axis color bars indicate the relative firing frequency (blue - low, red - high). ***Right:*** Distributions of minute-to-minute similarity values (at baseline, and following CFC) for the data shown in the FSMs. (**b**) CA1 network FSMs across the 5 longest bouts of NREM sleep in the 24 h following CFC.

Supplementary Figure 11

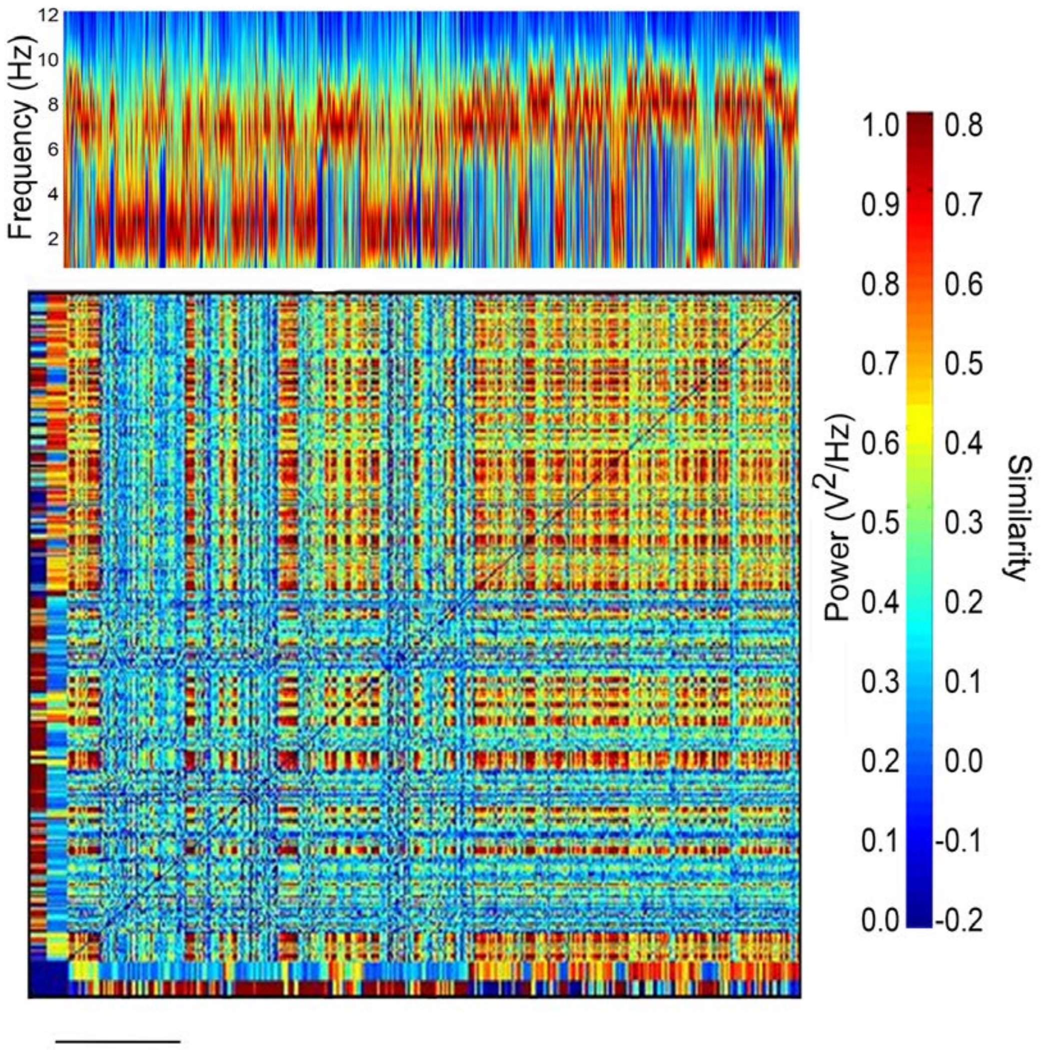

Supplementary Figure 11: Relationship between LFP spectral power and functional connectivity patterns in a representative mouse at baseline. Example of temporal relationships between CA1 LFP power (***top***) and FSM structure (***bottom***) over the same 6-h time period. Color in the body of the FSM denotes the degree of similarity between functional connectivity patterns at any given time point in the recording, and patterns at all other time points. Color bars along the outer edge of axes represents the behavioral state of the animal (blue - wake, red - NREM or REM, mixed sleep and wake are represented by color blends, *e.g.,*

yellow) and inner axis color bars indicate the relative firing frequency (blue - low, red - high). Scale bar = 1 h.

Supplementary Figure 12

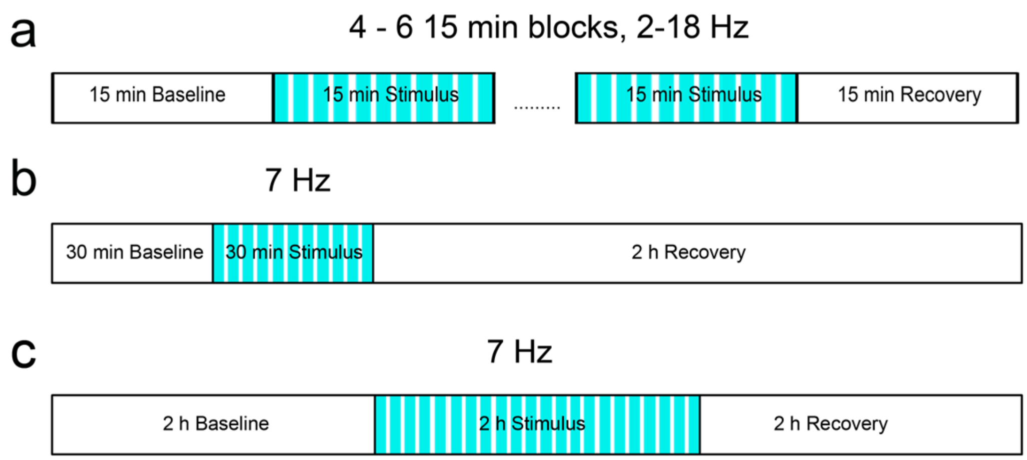

Supplementary Figure 12: Recording paradigms for optogenetic experiments. (**a**) PV+ interneurons in CA1 were stimulated at a range of frequencies in a random order (2-18 Hz, 40 ms pulses, 15 min for each frequency) for experiments described in **Fig. 8a-e**, and **Supplementary Fig.15a-b**. (**b**) CA1 neurons were recorded over a longer duration (30 min before and 2 h after 7 Hz stimulation) for experiments described in **Fig. 8f-h**, and **Supplementary Fig.15c-e**. (**c**) For experiments using non-anesthetized mice (**Supplementary Fig. 16**), CA1 neurons were recorded over a 2-h baseline, a 2-h period of 7 Hz stimulation, and a 2-h post-stimulation period.

Supplementary Figure 13

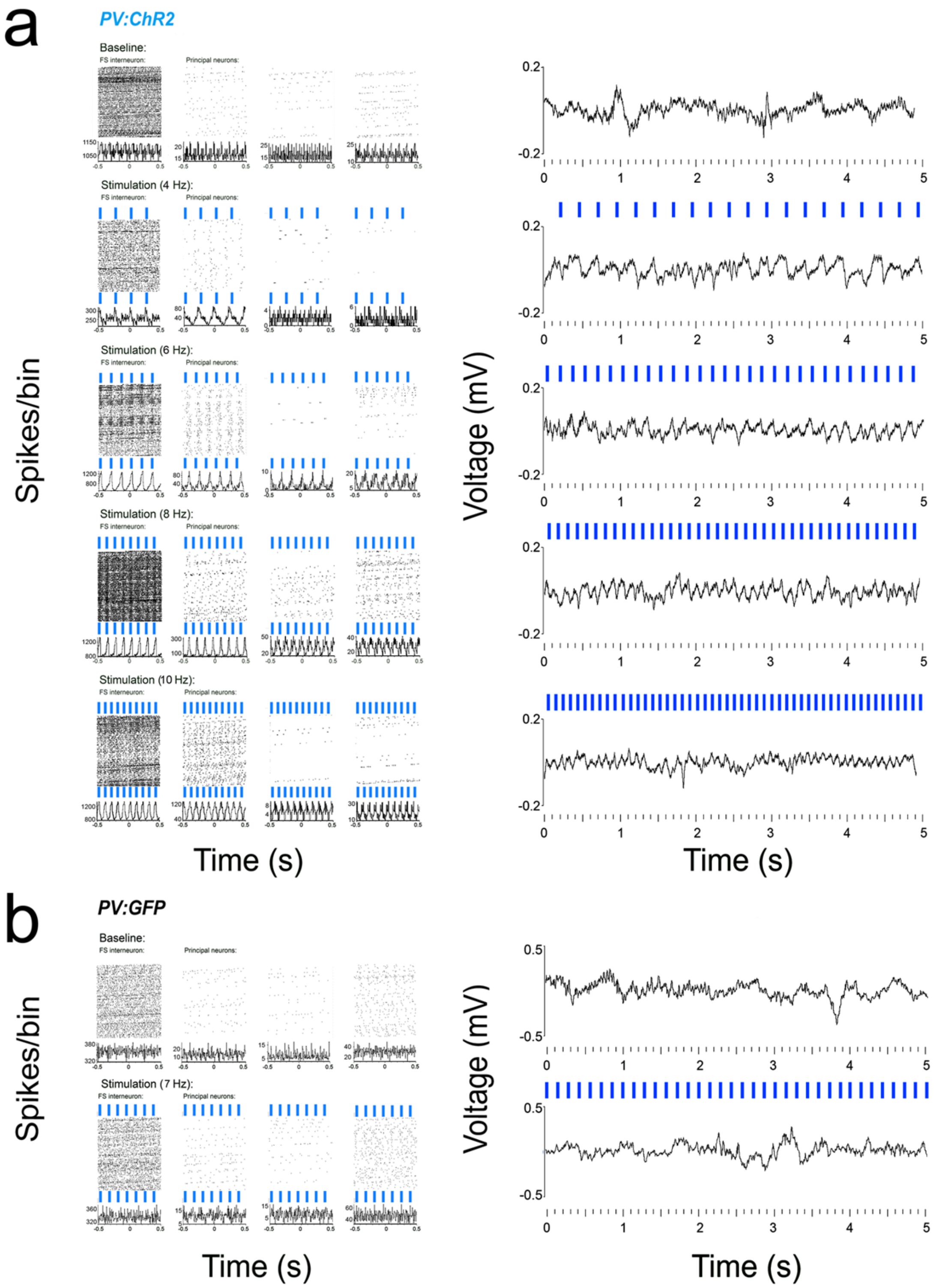

Supplementary Figure 13: Responses of CA1 neurons and LFPs across a range of optogenetic stimulation frequencies. (**a**) ***Left****:* Perievent firing rasters (top panels) and perievent firing histograms (bottom panels) for a representative CA1 FS interneuron and 3 neighboring principal neurons recorded from a *PV:ChR2* mouse, before and during rhythmic (4-10 Hz) stimulation of PV+ interneurons. ***Right****:* A representative 5-s LFP trace for one of the recording sites from in baseline and stimulation conditions. (**b**) ***Left****:* Perievent firing rasters (top panels) and perievent firing histograms (bottom panels) for a representative CA1 FS interneuron and 3 neighboring principal neurons recorded from a *PV:GFP* mouse, before and during rhythmic (7 Hz) stimulation of PV+ interneurons. ***Right****:* A representative 5-s LFP trace for one of the recording sites from in baseline and stimulation conditions.

Supplementary Figure 14

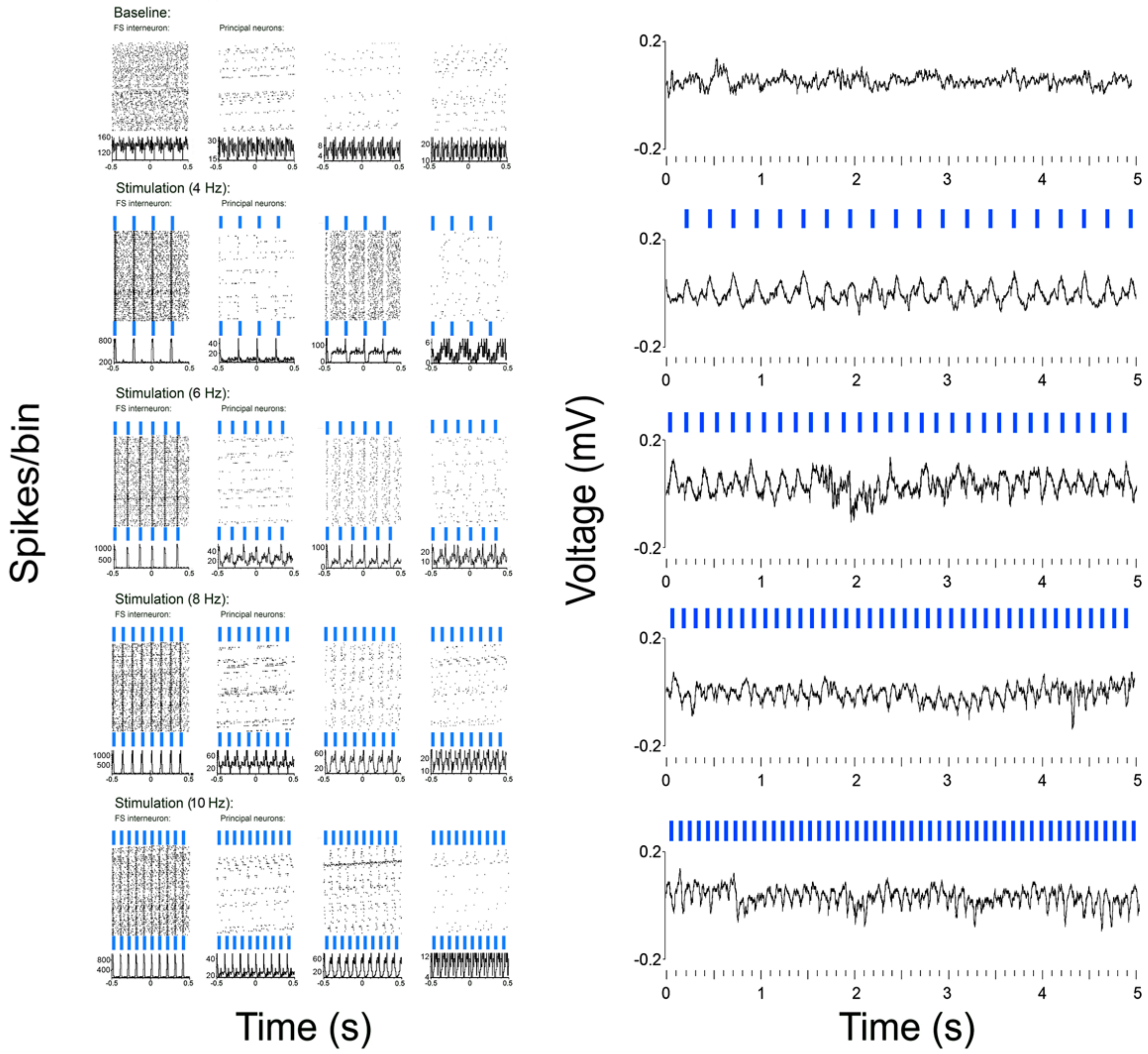

Supplementary Figure 14: Responses of CA1 neurons and LFPs across a range of optogenetic stimulation frequencies following viral transduction of PV+ interneurons with ChR2. ***Left:*** Perievent firing rasters (top panels) and perievent firing histograms (bottom panels) for a representative CA1 FS interneuron and 3 neighboring principal neurons recorded from a virally-transduced mouse, before and during rhythmic (4-10 Hz) stimulation of PV+

interneurons. ***Right:*** A representative 5-s LFP trace for one of the recording sites from in baseline and stimulation conditions.

Supplementary Figure 15

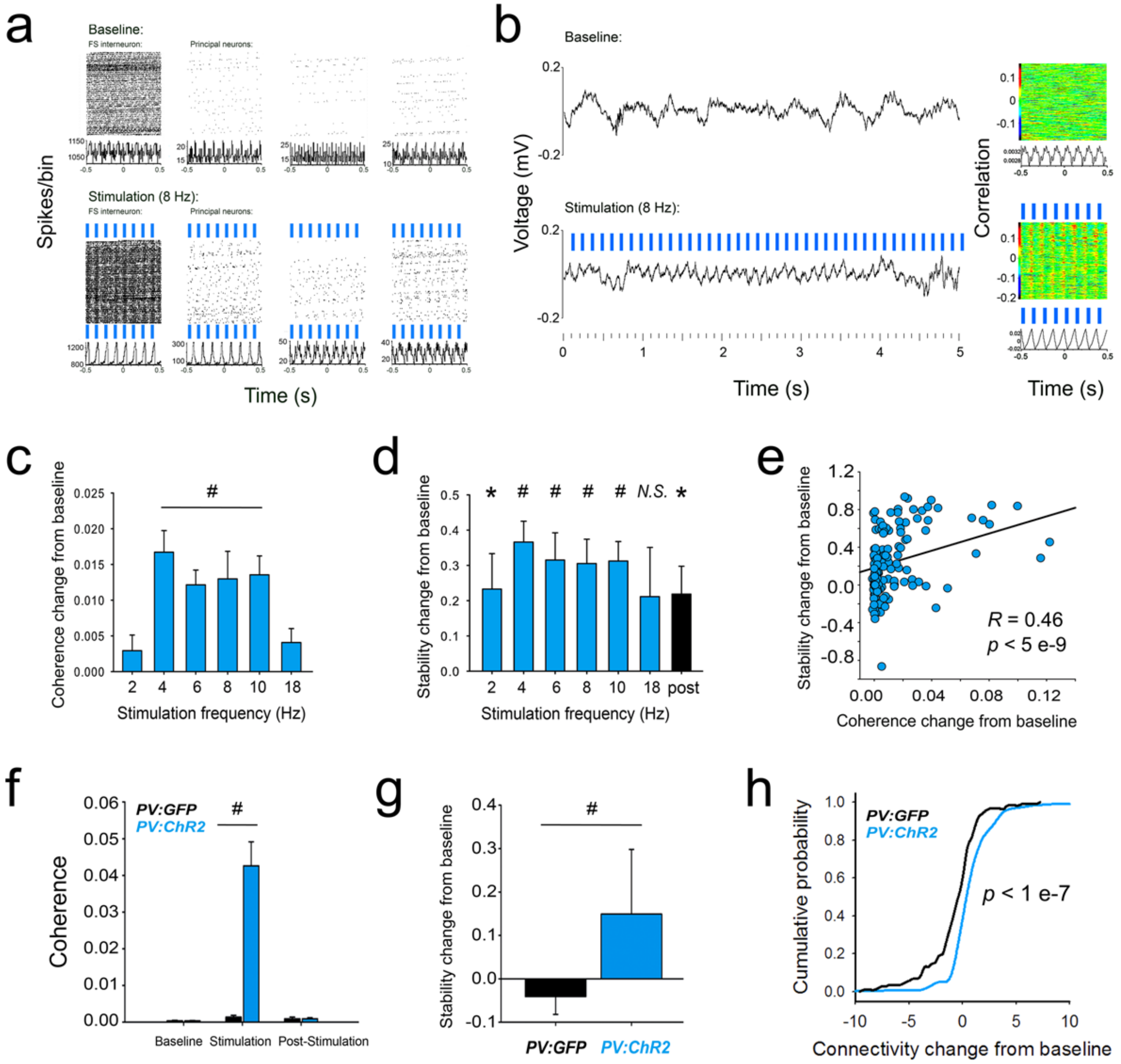

Supplementary Figure 15: Rhythmic optogenetic stimulation of PV+ interneurons increases CA1 network coherence, stability, and connection strength. (**a**) Perievent firing rasters (***top panels***) and perievent firing histograms (***bottom panels***) for a representative CA1 FS interneuron and 3 neighboring principal neurons recorded from a *PV:ChR2* transgenic mouse. Firing is shown over 250 s of recording before and during rhythmic (8 Hz) 473 nm light stimulation of PV+ interneurons. (**b**) A representative 5-s LFP trace (***left***) and perievent LFP

raster (***right***) for one of the recording sites from (**a**) in baseline and stimulation conditions. (**c**) Changes in spike-field coherence (from baseline) induced by various frequencies of rhythmic PV+ interneuron stimulation. (**d**) Changes in neuronal functional connectivity stability (from baseline) induced by various frequencies of rhythmic PV+ interneuron stimulation. (**e**) Across 4-10 Hz stimulation frequencies, changes in spike-field coherence predicted changes in stability of functional connectivity for individual neurons (Spearmann rank order, n = 153 neurons). (**f**) Comparison of CA1 spike-field coherence across a 30-min baseline period, 30 min of 7 Hz stimulation, and 2 h or post-stimulation recovery, in *PV:ChR2* (blue) and *PV:GFP* (black) mice. (**g**) Over the 2 h following 7 Hz stimulation, CA1 neuronal functional connectivity in *PV:ChR2* mice showed an increase in stability relative to baseline. For **c-g**, * indicates $p < 0.05$ and # indicates $p < 0.001$, Wilcoxon signed rank test. All values indicate mean ± SEM. (**h**) Neuronal functional connectivity strength also showed an increase in *PV:ChR2* mice relative to baseline. A similar change that was not seen in *PV:GFP* mice. p value indicates results of Kolmogorov-Smirnov test.

Supplementary Figure 16

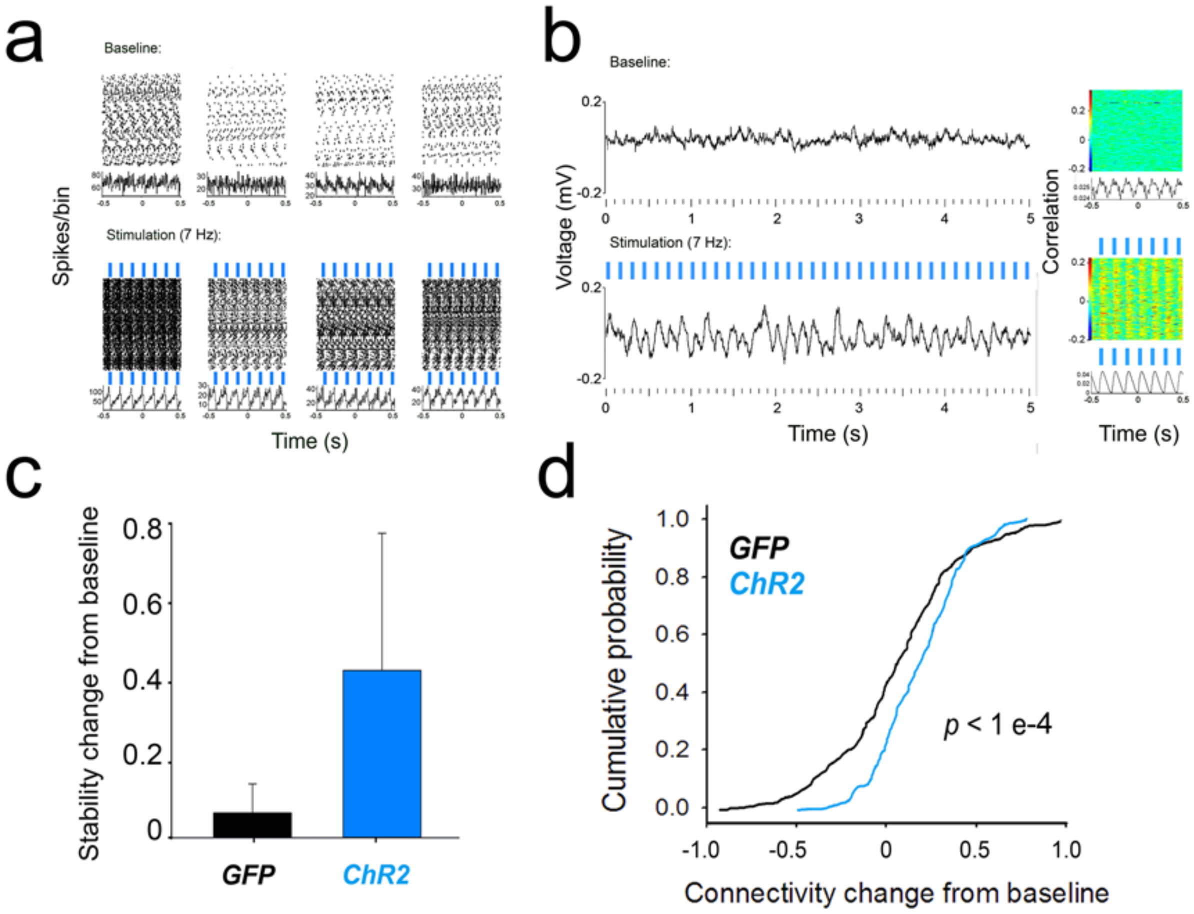

Supplementary Figure 16: Rhythmic optogenetic stimulation of ChR-2 expressing PV+ interneurons synchronizes firing and LFP rhythms in CA1 under non-anesthetized conditions. (**a**) Perievent firing rasters (***top panels***) and perievent firing histograms (***bottom panels***) for 4 representative CA1 principal neurons recorded from an awake, freely-behaving ChR2-transduced mouse. Firing is shown over 400 s of recording before and during rhythmic (7 Hz) 473 nm light stimulation of PV+ interneurons. (**b**) A representative 5-s LFP trace (***left***) and perievent LFP raster (***right***) for one of the recording sites from (**a**) in baseline and stimulation conditions. (**c**) Following a 2-h period of 7-Hz stimulation, CA1 neuronal functional connectivity in mice transduced with ChR2 showed a tendency for increased stability relative to baseline,

which was not seen in GFP expressing mice (*n* = 3 mice/group, *N.S.*, Wilcoxon signed rank test). Values indicate mean ± SEM. (**d**) Neuronal functional connectivity strength showed an increase in ChR2-transduced mice following 7 Hz stimulation, which was not seen in GFP-transduced mice. *p* value indicates results of Kolmogorov-Smirnov test, for *n* = 36 neurons (172 pairwise comparisons) recorded from ChR2-expressing mice, *n* = 32 neurons (144 pairwise comparisons) recorded from GFP-expressing mice.

Supplementary References:

1. Tricoire, L. *et al.* A Blueprint for the Spatiotemporal Origins of Mouse Hippocampal Interneuron Diversity. *J. Neurosci.* **31,** 10948–10970 (2011).

2. Lopez De Armentia, M. *et al.* Development/Plasticity/Repair cAMP Response Element-Binding Protein-Mediated Gene Expression Increases the Intrinsic Excitability of CA1 Pyramidal Neurons. *J. Neurosci.* **27,** 13909–13918 (2007).
